# Repositioning Lidocaine as an Anticancer Drug: The Role Beyond Anesthesia

**DOI:** 10.3389/fcell.2020.00565

**Published:** 2020-07-17

**Authors:** Daipeng Zhou, Lei Wang, Qingbin Cui, Ryma Iftikhar, Yanfei Xia, Peng Xu

**Affiliations:** ^1^Department of Anesthesiology, Pinghu First People’s Hospital, Jiaxing, China; ^2^College of Pharmacy and Health Sciences, St. John’s University, Queens, NY, United States; ^3^Department of Anesthesiology, Zhejiang Hospital, Hangzhou, China

**Keywords:** drug repositioning, lidocaine, chemosensitizer, anticancer, mechanisms

## Abstract

While cancer treatment has improved dramatically, it has also encountered many critical challenges, such as disease recurrence, metastasis, and drug resistance, making new drugs with novel mechanisms an urgent clinical need. The term “drug repositioning,” also known as old drugs for new uses, has emerged as one practical strategy to develop new anticancer drugs. Anesthetics have been widely used in surgical procedures to reduce the excruciating pain. Lidocaine, one of the most-used local anesthetics in clinical settings, has been found to show multi-activities, including potential in cancer treatment. Growing evidence shows that lidocaine may not only work as a chemosensitizer that sensitizes other conventional chemotherapeutics to certain resistant cancer cells, but also could suppress cancer cells growth by single use at different doses or concentrations. Lidocaine could suppress cancer cell growth *in vitro* and *in vivo* via multiple mechanisms, such as regulating epigenetic changes and promoting pro-apoptosis pathways, as well as regulating ABC transporters, metastasis, and angiogenesis, etc., providing valuable information for its further application in cancer treatment and for new drug discovery. In addition, lidocaine is now under clinical trials to treat certain types of cancer. In the current review, we summarize the research and analyze the underlying mechanisms, and address key issues in this area.

## Introduction

**FIGURE 1:**
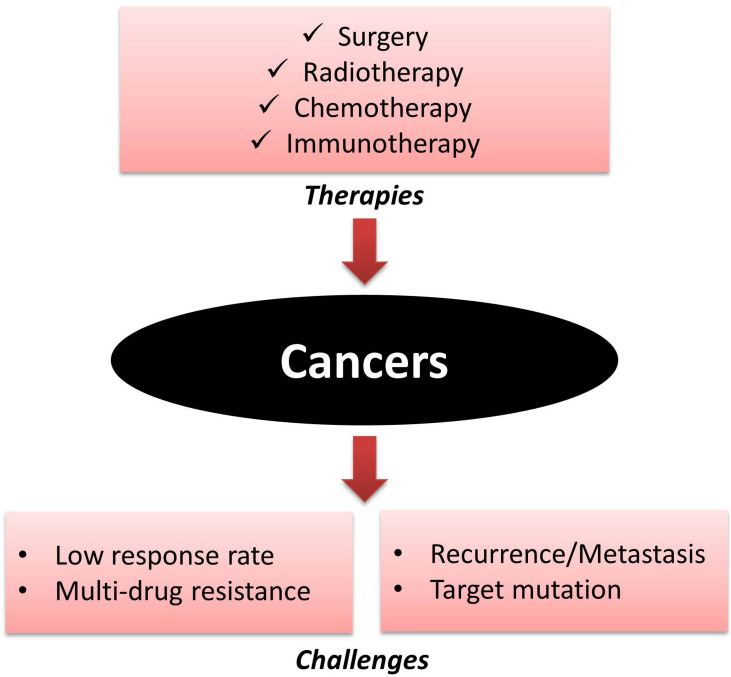
Cancer treatment and critical challenges.

Cancer treatments have made dramatic progress and achieved tremendous success for the last seven decades as a result of the development of conventional therapies, including surgery, chemotherapy, radiotherapy (DeVita and Chu, 2008), and innovative targeted small-molecule tyrosine kinase inhibitors (Prasad et al., 2016; Lee et al., 2018), as well as the cutting-edge immunotherapy (Macchiarulo, 2017; Harjes, 2018). The quality of life of cancer patients has improved significantly by precisely eliminating cancer cells without harming normal cells (Re et al., 2018; Salgado et al., 2018). However, many challenges remain to be solved (Figure 1). Examples include the low response rate, even in those patients with positive tyrosine kinases mutations or with programmed cell death 1 (PD-1) and programmed death ligand 1 (PD-L1) (Prasad et al., 2016; Sambi et al., 2019). Other challenges include cancer recurrence and target mutations, as well as the skyrocketing financial pressure, etc. Another major ominous issue in cancer chemotherapy is multi-drug resistance (MDR), which is harnessed by cancer cells to become resistant to various chemotherapeutics (Pavan et al., 2014; Riganti et al., 2015). Various strategies have been developed to address these challenges (Bode and Dong, 2018; Cui et al., 2018a, 2019b; Karlovich and Williams, 2019; Wise and Solit, 2019). Generations of selective chemotherapeutics and monoclonal antibodies have been developed to improve the response rate, and diminish adverse effects (DeVita and Chu, 2008; Palazzo et al., 2010; Mittra and Moscow, 2019). MDR can be induced by many factors, such as the activated defensive system which eliminates reactive oxygen species (ROS) caused by various chemotherapeutics (Cui et al., 2018a); the overexpression of ATP-binding cassette (ABC) transporters which transport their substrates, including many anticancer drugs via a mechanism known as efflux to reduce the concentrations of various chemotherapeutics (Li et al., 2016; Mottino, 2019); and the provoked DNA repair to become resistance to antimetabolite and certain tyrosine kinase inhibitors (TKIs) (Nikitaki et al., 2015; Sun et al., 2018), as well as up-regulated anti-apoptotic enzymes (Adams et al., 2018), etc. Each of these mechanisms could be countered (totally or partially) by specific strategies, such as drug combinations, although the drug–drug interactions and further drug resistance are still unavoidable. Novel anticancer agents remain unmet clinical needs.

Other novel anticancer drugs discovery strategies include the old drugs repositioning (Pushpakom et al., 2018). Drug repositioning, also known as old drugs for new uses, is a strategy to identify the novel application of approved drugs that have been assessed for safety and efficacy (to their original indications) (Serafin et al., 2019), resulting in low-cost and low-risk new drug development. Successful examples in preclinical/clinical trials include the combination of conventional chemotherapeutics with certain TKIs (Fan et al., 2018; Cui et al., 2019a, Wang et al., 2020), phosphodiesterase-5 inhibitors (Tiwari and Chen, 2013), and natural products, such as supplemental flavonoids (Cui et al., 2018b; Ye Q. et al., 2019), at various concentrations, to overcome certain MDR mediated by ABC transporters. Recently, local anesthetics and their applications in cancer treatment have attracted much interest (Nowacka and Borczyk, 2019; Yap et al., 2019). These compounds are found to work either as chemo-sensitizers, or by single use as cancer cell growth suppressor. In this review, we would like to summarize and discuss those studies of lidocaine, one of the most commonly used local anesthetics in clinical settings, and focus on its applications and mechanisms in cancer treatment.

## Lidocaine in Cancer Treatment

Originally, anesthetics are used to suppress the excruciating pain during various surgeries. Meanwhile, these anesthetics also possess other functions, examples include lidocaine. As it has been shown that through different mechanisms, such as sodium channel inhibitor and the regulation of G protein-coupled receptors, lidocaine exerts its multifunctional effects in anti-inflammation (Caracas et al., 2009; Cruz et al., 2017), analgesia and antihyperalgesia (Tian et al., 2009), immuno-modulation (Kim et al., 2017), anti-arrhythmia (Harrison and Collinsworth, 1974), and as an antibacterial (Begec et al., 2007). Importantly, as the focus of current review, growing evidence suggests that local anesthetics may also provide benefits in the treatment of cancer by inhibiting proliferation, invasion, and migration of certain cancer cells (Grandhi and Perona, 2019). Literatures show that lidocaine is not only able to kill various types of cancer cells, but has also been reported to sensitize various chemotherapeutics to many types of resistant cancer cells.

### Lidocaine Sensitizes Cisplatin in Inducing Apoptosis via Multiple Pathways

A recent study demonstrated the sensitizing effects of lidocaine to cisplatin on human breast cancer lines MCF-7 and MDA-MB-231, a highly aggressive triple-negative breast cancer (TNBC) cell line that exhibits certain MDR properties (Koh et al., 2019*;* Thi-Kim et al., 2019). As shown in this research, lidocaine (0.01–1 mM), when combined with cisplatin, significantly enhanced the apoptosis ratio and the inhibitory effects of cisplatin on MCF-7 and MDA-MB-231 cells. The combination also suppressed the colony formation of MCF-7 and MDA-MB-231 cells. The results of combination therapy were much better than single administration of either lidocaine or cisplatin. The Western blot assay indicated, after co-treatment with lidocaine and cisplatin, higher expression of activated caspase-3 that regulated apoptosis, cleaved poly (ADP-ribose) polymerase (PARP) that promoted DNA repair, and cytochrome c (apoptosis modulator) was released from the mitochondria in MCF-7 cells were induced. Mechanistic studies revealed that lidocaine could suppress the methylation of certain genes, such as retinoic acid receptor β2 (RARβ2) and Ras association domain family 1 isoform A (RASSF1A), two promoters of tumor-suppressive genes. These findings in the present study indicated that, via suppression of RARβ2 and RASSF1A methylation, lidocaine sensitized cisplatin to breast cancer cells (Li et al., 2014).

Overexpression of miR-21 could regulate the phosphatase and tensin homolog (PTEN)/phosphoinositide-3-kinase (PI3K)/protein kinase B (PKB, or AKT) and programmed cell death 4 (PDCD4)/c-Jun N-terminal kinase (JNK) pathways, which are pivotal to cancer cells growth in cisplatin-resistant non-small cells lung cancer (NSCLC) A549/DDP cells (Ren et al., 2016). A study found that lidocaine alleviated cisplatin resistance and exhibited good inhibitory effects on the cell viability and cell migration and invasion at 10 μM. Mechanistic study indicated that the inhibitory effects were attributed to the induction of remarkable apoptosis by down-regulating mi-R-21 on A549/DDP cells—similar results as mi-R-21 inhibitor. The inhibition of mi-R-21 induced by lidocaine finally lead to the suppression of PTEN/PI3K/AKT and PDCD4/JNK pathways, leading to the resensitizing of cisplatin to A549/DDP cells (Yang Q. et al., 2019).

Multi-metastasis is one of the leading causes that result in mortality among breast cancer patients (Medeiros and Allan, 2019). Freeman et al. set out to determine the metastasis upon treatment of lidocaine/cisplatin by single or combinationary use in 4T1 cells xenograft mice model. Their results indicated that, compared to the control and cisplatin groups, the number of lung metastasis colony count was significantly reduced by the combination of cisplatin (3 mg/kg) and lidocaine (1.5 mg/kg); meanwhile, it didn’t impact the vascular endothelial growth factor (VEGF), a factor that is closely involved in cancer growth and migration (Eroglu et al., 2017), and the level of interleukin-6 (IL-6), an pro-inflammatory cytokine that promotes migration (He et al., 2018), as tested in serum. This study provided evidence that lidocaine might suppress metastasis in a breast cancer model, warranting further study for the underlying acting mechanisms and further evaluation in preclinical trials (Freeman et al., 2018).

Given its ability in sensitizing cisplatin in the cancer treatment, certain pharmaceutical formulations have been developed to achieve the co-delivery of lidocaine and cisplatin, including nanoparticles that possess well-controlled loading capacities and cancer-targeting properties (Swain et al., 2016; Wen et al., 2017). One nanogel system modified on cRGDfk, a peptide segment-cRGDfk with high affinity to α_v_β_3_ integrin, was designed by Gao et al. and tested in chemo-resistant MDA-MB-231 breast cancer cells. This nanogel system (optimized concentration of 10 μM cisplatin and 50 μM lidocaine) demonstrated enhanced cytotoxic effects toward MDA-MB-231 cells by inducing substantial apoptosis *in vitro*. The combination therapy suppressed the metastasis of MDA-MB-231 breast cancer cells in xenograft mouse model with 6 mg/kg of cisplatin and 24 mg/kg of lidocaine via intravenous administration. In addition, this nanogel system showed a favorable safety profile, as it alleviated body-weight loss upon single use of cisplatin. More importantly, this nanogel system also succeeded in targeting accumulation in tumor tissues, as well as in the inhibition of tumor growth. The nanogel system provided a new therapy regimen for the treatment of breast cancer that overexpressed α_v_β_3_ integrin (Gao et al., 2018).

### Lidocaine Sensitizes 5-Fluorouracil

Lidocaine also demonstrates sensitizing effects toward other chemotherapeutics, such as 5-fluorouracil (5-FU) (Wang et al., 2017) and mitomycin C (Yang X. et al., 2018).

In a study conducted by Wang et al. on the SK-MEL-2 melanoma cell line, although lidocaine (0*–*100 μM) exert limited toxic effects, its combination with 5-FU significantly enhanced the anticancer potency and apoptosis induction effects of 5-FU. Further mechanistic studies revealed that the overexpression of miR-493, and its target gene, *Sox4*, alone with the decreased levels of phosphined PI3K, AKT, and Smad2/3, conferred the re-sensitizing of SK-MEL-2 cells to 5-FU by lidocaine. This research indicated that lidocaine sensitized melanoma cells to 5-FU via the up-regulation of miR-493 and the down-regulation of Sox4-mediated PI3K/AKT and Smad pathways (Wang et al., 2017).

Recently, Zhang et al. reported that lidocaine might regulate ABC transporters proteins, leading to the sensitizing of 5-FU to choriocarcinoma cells (Zhang et al., 2019). As shown in this study, lidocaine (10–1,000 μM) exhibited limited toxic effects to human choriocarcinoma JEG-3 and JAR cells (with only moderate cytotoxic effects at 1,000 μM), while when combined (1, 10, and 100 μM) with 5-FU at non-toxic concentrations, it enhanced the sensitivity of 5-FU to these two cell lines via increased induction of apoptosis. Importantly, lidocaine down-regulated the levels of breast cancer resistance protein (BCRP or ABCG2), P-gp, multidrug resistance-associated protein 1 (MRP1), and MRP2 in cancer cells, probably via the inactivation of PI3K/AKT pathway, as confirmed by Western blot analysis. The overexpression of ABCG2 could antagonize the lidocaine induced sensitizing effects and inactivated PI3K/AKT pathway (Zhang et al., 2019), indicating 5-FU to be a substrate of ABCG2, and lidocaine might impact the efflux function of ABCG2 (Tang et al., 2017). This study provided interesting information for the reversal of MDR mediated by ABC transporters (Li et al., 2016*;* Ji et al., 2019) by lidocaine, indicating its ability to reverse the resistance of certain chemotherapeutics that are substrates of ABC transporters and warranting further research.

### Lidocaine Sensitizes Mitomycin C and Pirarubicin Without Showing Obvious Adverse Effects

Yang et al. evaluated the combination of lidocaine with other anticancer drugs, such as mitomycin C, pirarubicin, and Su Fu’ning lotion (an approved traditional Chinese medicine in China) on BIU-87 human bladder cancer cells and in a tumor-bearing mouse model. The results indicated lidocaine (1.25, 2.5, or 5 mg/mL, or 5.3, 10.6 or 21.3 μM) was a chemosensitizer as it enhanced the cytotoxicities of all three anticancer drugs, among which, the combination of lidocaine with mitomycin C was the most potent. In the *in vivo* test, this optimized combination exhibited lower body-weight loss and higher survival rates *in vivo* via intravesical administration compared to mitomycin C alone without showing any evidence of toxicity, suggesting a safe and promising therapy for the treatment of bladder cancer and warranting further research for the exact mechanisms (Yang X. et al., 2018).

### Lidocaine Sensitizes Hyperthermia Therapy via Regulating Cell Cycle and Heat Shock Proteins

Lidocaine was found to regulate the induction of heat shock proteins (HSPs) (Senisterra and Lepock, 2000), which could be applied in hyperthermia therapy. Raff et al. showed that in an *in vitro* study, lidocaine, at different concentrations (ranged from 0–0.3%), when combined with hyperthermia, exhibited selectivity to skin cancer cell lines and mucosal cancer cell line, such as human melanoma cells A375, murine basal cell carcinoma ASZ, and human cervical cancer cell line HeLa, over normal human keratinocytes (KertR) cells and human foreskin fibroblasts (HFF1). The combination treatment, 42°C of hyperthermia combined with 0.1–0.2% lidocaine, significantly inhibited the proliferation of cancer cell lines via cell arrest induction in S-phase, indicating the combination to be a promising regimen for selective killing of skin cancer cells (Raff et al., 2019).

The research above suggested that lidocaine (ranged from 1–100 μM) could work as a chemosensitizer to enhance the sensitivity of cisplatin, 5-FU, and mitomycin C. Its full potential remains to be explored and warrants further preclinical/clinical trials of these combination.

### Lidocaine Suppresses Cancer Growth

Lidocaine not only works as a chemosensitizer; it may also exert inhibitory effects toward various cancer cells and in tumor xenograft models by single use at higher concentrations.

#### Lung Cancer

Lung cancer, categorized into two main subtypes, small-cell lung cancer (SCLC) and NSCLC, is the primary cause of cancer-related death worldwide (Caballero et al., 2018*;* Arenberg, 2019). Lidocaine has been found to kill lung cancer cells *in vitro* and *in vivo*.

Beck-Schimmer’s group confirmed that certain amide-type local anesthetics, including lidocaine, could inhibit cancer cells by suppressing tumor necrosis factor alpha (TNFα)-induced Src activation, a mechanism related to its anti-inflammatory effects (Piegeler et al., 2015). Further study showed that lidocaine (1 nM to 100 μM, at clinically relevant concentrations) remarkably suppressed the activation/phosphorylation of AKT, focal adhesion kinase (FAK), and caveolin-1 induced by TNFα in lung adenocarcinoma NCI-H838 cells. Matrix-metalloproteinase 9 (MMP-9) is an enzyme that catalyzes the degradation of the extracellular matrix and basal lamina to allow migrating cancer cells to escape the primary tumor site, or to locate to a new spot to form the satellite lesions (El-Badrawy et al., 2014*;* Lee et al., 2015), which is important to from new solid tumors. Lidocaine significantly suppressed the MMP-9 secretion as confirmed by the immunosorbent assay, and it completely inhibited the cancer cell invasion, indicating its potential in preventing cancer invasion and metastasis (Piegeler et al., 2015). Another local anesthetic ropivacaine exhibited similar efficacy but at lower concentrations compared to lidocaine as shown in this study (Piegeler et al., 2015), which necessitates a broad screening of these type anesthetics.

To reveal more details on the mechanisms of the anticancer effects of lidocaine in lung cancer, Zhang et al. checked the related proteins by real-time quantitative PCR (qPCR) and Western blot assay in the samples of lung cancer patients. They confirmed that Golgi phosphor protein 2 (GOLPH2), a Golgi phosphoprotein that had been described as a potential serum marker of hepatocellular carcinoma, prostate cancer, and renal cell cancer (Duan et al., 2018; Li H. et al., 2018), was overexpressed in the patients with higher grade malignancy. Lidocaine (0.5, 2, and 8 mM, which were determined by MTT and BrdU assays) was found to suppress the expression of GOLPH2 in NSCLC A549 cells, leading to cell cycle arrest at the G1 phase and cell proliferation inhibition (Zhang et al., 2017).

In addition, lidocaine showed activity in suppressing postoperative atrial fibrillation (POAF), which occurs frequently after lung cancer surgery (Akkus and Oner, 2019), indicating its broad potent applications in lung cancer treatment.

#### Breast Cancer

Breast cancer is the main cause of cancer-related deaths among females, accounting for approximately 7% of female cancer deaths due to uncontrolled metastasis (Anders et al., 2009; Vogel et al., 2011). Lidocaine exhibited activities in suppressing breast cancer growth.

C-X-C motif chemokine ligand 12 (CXCL12), and its receptor CXCR4, are proteins that regulate physiological and pathological processes, such as embryogenesis, angiogenesis, and inflammation (*Del et al., 2018;* Peled et al., 2018). Both proteins were found to be overexpressed in breast cancer (Wu et al., 2015; Smolkova et al., 2016). D’Agostino et al. found that, lidocaine, at 10 and 100 μM, reduced the motility of human breast cancer MDA-MB-231 cells as measured by the scratch wound assay and chemotaxis experiments. Further study indicated that the effects were mediated by the suppression of CXCL12/CXCR4 signaling, leading to the down-regulation of calcium (Ca^2+^) release and collapsed cytoskeleton remodeling, providing mechanistic insights of lidocaine in inhibiting cancer cell migration (D’Agostino et al., 2018).

Other mechanisms of metastasis inhibition induced by lidocaine in breast cancer cells might be attributed to its anti-inflammatory and anti-angiogenic effects according to a study in 4T1 breast cancer cell line *in vitro* and *in vivo* (Johnson et al., 2018). In this study, lidocaine (1.5 mg/kg, intravenous, followed by 25 min infusion at 2 mg/kg/h) showed no growth inhibition in tumor diameter, but it reduced the number of colony counts in lung and liver metastasis significantly via the inhibition of pro-inflammatory and angiogenic cytokine expression as tested in serum in animal models (Johnson et al., 2018). Similar results and mechanisms were also observed in Freeman et al.’s study (Freeman et al., 2019), indicating lidocaine’s role in suppressing metastasis of breast cancer via the suppression of pro-inflammation factors.

Given lidocaine’s effect in suppressing metastasis, its further application in various breast cancer cell lines, including normal breast epithelial cells MCF-10A, luminal breast cancer cell MCF-7, TNBC MDA-MB-231, and SKBr3 human epidermal growth factor receptor 2 (HER2) positive cells and in MDA-MB-231 cells xenograft model at clinically relevant concentrations were studied (Chamaraux-Tran et al., 2018). Lidocaine (0.1–10 mM determined by MTT assay) showed selectivity in suppressing the viability and migration of cancer cells over normal cells. Under clinically relevant dose for analgesia (100 mg/kg as adjusted according to the body surface area normalization method, injected intraperitoneally), lidocaine enhanced the survival rates in mice models of breast cancers (Chamaraux-Tran et al., 2018), warranting further preclinical/clinical studies.

Lirk et al. (2012) found that lidocaine (0.01, 0.1, and 1 mM) could demethylate DNA in both estrogen receptor (ER)-positive and -negative breast cancer cell lines (BT-20 and MCF-7 cells). This demethylation of DNA could finally lead to the inhibition of tumor growth and also provoke certain tumor suppressors, such as Ras association domain family 1 isoform A (RASSF1A), glutathione S-Transferase pi 1 (GSTP1), and myogenic differentiation 1 (MYOD1). Furthermore, the combination of lidocaine with another anticancer drug, 5-aza-2′-deoxycytidine, exerted synergistic demethylating effects (Lirk et al., 2014), suggesting that lidocaine regulates epigenetics—an underlying mechanism for its ability in suppressing tumor growth.

#### Liver Cancer

Liver cancer, also known as hepatic cancer, is a type of cancer that starts in the liver. Liver cancer ranks as the sixth-most frequent cancer, and it’s one of the most rapidly progressing types of cancer. Lidocaine was also found to kill liver cancer cells.

Juri et al. examined and confirmed the inhibitory efficacies of lidocaine in the proliferation of human hepatocarcinoma HepG2 cells and normal liver fibroblasts LX2 cells. Their results showed that lidocaine (0.5–3 μM) could concentration-dependently suppress the cell viability of both cells, while it showed stronger inhibition to HepG2 cells, suggesting a selectivity profile. Mechanistic studies indicated that lidocaine down-regulated the expression of anti-apoptotic p53 in HepG2 cells, but not in LX2 cells (Jurj et al., 2017).

Another recent study conducted *in vitro* and *in vivo* by Xing et al. further confirmed lidocaine’s (0.1–10 mM) effects in inhibiting HepG2 cells growth (Xing et al., 2017) by inducing apoptosis through increasing the Bax/Bcl-2 ratio, an initial step in apoptosis activation (Adams et al., 2018), and activating caspase-3 via ERK1/2 and p38 pathways. Their mechanistic studies showed that lidocaine exerted its anticancer effects via cell cycle arrest at G0/G1 phase. More importantly, lidocaine not only reduced the tumor growth (30 mk/kg) through intraperitoneal injection, but also sensitized cisplatin in this animal study, indicating a combination therapy in treating hepatocellular carcinoma without exhibiting any obvious toxic effects (Xing et al., 2017).

Liu et al. (2018) evaluated the level of cytoplasmic polyadenylation element binding protein 3 (CPEB3) in liver cancer patients’ samples. They found that CPEB3 was down-regulated, leading to poor prognosis and high-grade malignancy (Liu et al., 2018). An *in vitro* study showed that lidocaine (0.1, 0.2, 0.5, 1, 2, 5, and 10 mM, as determined by cell counting kit-8 reagent) could up-regulate CPEB3 (at both mRNA and protein level), leading to the inhibition of proliferation and colony formation of HepG2 cells (Liu et al., 2018).

#### Gastric Cancer

Gastric cancer ranks as the third leading reason for cancer-related deaths (Johnston and Beckman, 2019), with more than 1 million people newly diagnosed worldwide each year, causing a huge burden (Thrift and El-Serag, 2019). As one of the most-used local anesthetics in gastric cancer surgery, lidocaine also inhibits the proliferation of gastric cancer cells.

As shown in Sui’s study, lidocaine (1, 5, and 10 mM as determined by cell counting kit-8 reagent) suppressed the viability, migration, and invasion of gastric cancer MKN45 cells, meanwhile it induced more apoptosis. Further studies indicated that these effects were mediated, at least partially, by the up-regulation of miR-145, an important regulator of gastric cancer, which in turn inactivated the mitogen-activated protein kinase (MAPK)/ERK and nuclear factor kappa-light-chain-enhancer of activated B cells (NF-κB) pathways. Transfection of miR-145 inhibitor showed contrary effects on the same cell line as compared to lidocaine, confirming a miR-145-mediated mechanism (Sui et al., 2019).

Another study conducted on human gastric cancer SGC7901 and BGC823 cells, lidocaine inhibited their proliferation, migration, and invasion and induced apoptosis in a concentration- (0.1–10 Mm, determined by MTT) and time- (0–72 h) dependent manner. Mechanistic studies showed that lidocaine increased the Bax/Bcl-2 ratio, increased the anti-apoptotic p-p38, and altered the MAPK expression, suggesting a mitochondria-mediated apoptosis pathway (Ye L. et al., 2019).

On gastric cancer cells AGS and HGC-27 cells, lidocaine (10 μM) suppressed the proliferation and migration, while it exerted no invasion inhibitory effects on both cancer cells. ERK1/2, key kinases playing pivotal roles in speeding the gastric cancer cells proliferation (Ahn et al., 2017), were down-regulated by lidocaine (Yang W. et al., 2018). Interestingly, another local anesthetic, ropivacaine was also found to possess similar efficacy as lidocaine as shown in this study (Yang W. et al., 2018).

Lidocaine not only worked in suppressing gastric cancer growth; it also exhibited potential in relieving persistent hiccups caused by gastric cancer (Kaneishi and Kawabata, 2013), suggesting its broad application in the treatment of gastric cancer and gastric cancer-related diseases/syndromes.

#### Colorectal Cancer

Colorectal cancer, a type of cancer that develops in the colon or rectum, is more common in developed countries than in developing countries, possibly due to old age and different lifestyles, with more than 1 million new diagnosed cases worldwide each year as of recent (Siegel et al., 2017).

Qu et al. (2018) found that lidocaine (0.5, 1 mM, as determined by cell-counting kit-8 reagent) exhibited antiproliferative effects on colorectal cancer SW480 and HCT116 cells by inducing apoptosis via the suppression of epidermal growth factor receptor (EGFR) through upregulation of miR-520a-3p, whose target was EGFR. In addition, lidocaine (0.5 mM) could reverse miR-520a-3p reduction caused by miR-520a-3p inhibitor, confirming its mechanism involving with miR-520a-3p (Qu et al., 2018).

A recent study showed that lidocaine inhibited the growth of colon cancer cells lines HCT-116 (948.6 μM) and RKO (2,197 μM) cells via regulating the activation of apoptosis proteins, such as caspase-8, p53 protein, and survivin, as well as HSP-27 and HSP-60 (Tat et al., 2019).

Another study conducted in HT-29 and SW480 colon carcinoma cell lines, lidocaine (1 mM as determined by BrdU labeling solution) induced cell cycle arrest, but it showed no significant proliferation inhibition or apoptosis on both cell lines (Bundscherer et al., 2017).

#### Melanoma

Melanoma is the most serious type of skin cancer and the leading cause of skin cancer related deaths, which can also be treated by certain concentrations of lidocaine.

Kang et al. (2016) found that lidocaine (2%), ropivacaine (0.75%), and their combination exhibited cytotoxic effects to melanoma cell lines A375 and Hs294T cells via the induction of apoptosis, and this effect was mediated through the activation of caspase-3 and caspase-8.

Another study conducted by Chen et al. showed that lidocaine (0.1, 3, and 5 mM, as determined by cell-counting kit-8 reagent) could kill A375 melanoma cells and suppress the colony formation in a concentration-dependent manner via cell cycle arrest at G1 phase (Chen et al., 2019). These effects were mediated by the down-regulation of Ki-67, a protein associated with cell proliferation and the cell division cycle (Robinson et al., 2018; Su and Chen, 2019), and down-regulation of phosphorylation of ERK. Importantly, lidocaine, via intravenous injections, significantly reduced the tumor volumes and weights in A375 cells xenograft mice model, indicating it a potential agent in combating melanoma (Chen et al., 2019).

Gao et al. examined the *in vitro* and *in vivo* effects of lidocaine on B16 melanoma cells. The results showed that lidocaine (50–200 μM, which were clinically relevant) inhibited cancer cells growth by inducing apoptosis. Mechanistic studies indicated that lidocaine cut the connections of matrigel and capillary formation, down-regulated vascular endothelial growth factor (VEGF)-activated VEGF receptor 2 (VEGFR2) phosphorylation, phospholipase Cγ (PLCγ)-protein kinase C (PKC)-MAPK, and FAK-paxillin in endothelial cells, leading to the inhibition of angiogenesis and cancer cells migration. Importantly, lidocaine (30 mg/kg), through intraperitoneal injection, significantly suppressed the angiogenesis and tumor growth in an animal model, suggesting its potential in treating melanoma by working as an angiogenesis inhibitor (Gao et al., 2019).

In addition, lidocaine (1.5 mg/kg) shortened the recovery of skin damage due to melanoma surgery, accelerated the wound healing, relieved the pain, and promoted the postoperative skin reconstruction (Xu et al., 2019), suggesting its potential in treating melanoma-related skin diseases.

#### Glioma

Malignant glioma is a common brain cancer with limited therapy and terrible prognosis (Seliger and Hau, 2018). Lidocaine has been shown to kill glioma cells via the suppression of the transient receptor potential melastatin 7 (TRPM7), as shown in Leng et al.’s research (Leng et al., 2017). TRPM7, a subfamily of TRPM family proteins, regulates the entry of Ca^2+^ and Mg^2+^. It is a hallmark of many cancers, including head and neck cancer, ovarian cancer, prostate cancer, and pancreatic cancer, playing a key role in cell function and cell cycle (Zou et al., 2019). It is also a promising target in treating malignant glioma (Leng et al., 2015). Leng et al. (2017) found that lidocaine (1, 3 mM) inhibited 30–50% TRPM7 expression and suppressed the proliferation of rat C6 glioma cells and human A172 glioblastoma cells. Another study also showed that lidocaine (0–40 mM) might correlate with transient receptor potential cation channel subfamily V member 1 (TRPV1), leading to induction of apoptosis of U87-MG glioma cells via an increase of intracellular Ca^2+^ concentration and regulation of mitochondrial membrane potential (Lu et al., 2016).

A similar mechanism was also confirmed by Jiang et al., as they found that lidocaine (10 μM, 100 μM, and 1 mM) could regulate transient receptor potential cation channel subfamily V member 6 (TRPV6) in MDA-MB-231 cells, PC-3 prostatic cancer cells, and ES-2 ovarian cancer cells. Lidocaine, at lower-than-clinical concentrations, suppressed the invasion and migration of all three cancer cell lines. Lidocaine down-regulated the mRNA level and protein expression of TRPV6, leading to a reduction of Ca^2+^ influx (Jiang et al., 2016).

These studies suggested that lidocaine, as a voltage-gated Na(+) channel inhibitor, may exert its anticancer effects via regulating other cation channels.

#### Tongue Cancer

Tongue cancer is a type of head and neck cancer that can be caused by smoking, drinking alcohol, and infection with the human papillomavirus (HPV) or Epstein-Barr virus (EBV) (Jiang et al., 2015).

In EGFR-overexpressing tongue cancer cell line, CAL27 cells, lidocaine suppressed the EGF-stimulated EGFR activity and inhibited the proliferation of CAL27 cells. This study suggested that by administering topically within the oral cavity, lidocaine cold suppress the proliferation of human tongue cancer cells (Sakaguchi et al., 2006). A recent study also confirmed the suppressing effects of lidocaine to the cell migration, growth, and survival of esophageal carcinoma at >100 μM, which was mediated by inhibiting mitochondrial respiration (Zhu et al., 2020).

## Discussion and Future Perspectives

The studies above indicate that lidocaine, besides its original anesthetic effect, could also act as an anticancer agent.

**FIGURE 2:**
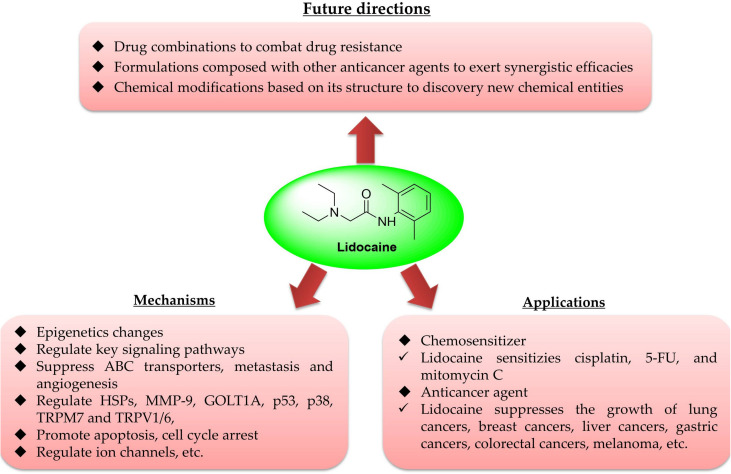
Overview of lidocaine in cancer treatment.

First, lidocaine could serve as a chemosensitizer, as it could markedly enhance the cytotoxicity of cisplatin in various cancer cells. Furthermore, lidocaine can sensitize 5-FU, cytomycin C, as well as the hyperthermia therapy, etc., to certain cancers *in vitro* and *in vivo*. Second, lidocaine had broad anticancer spectrum by single administration at various doses or concentrations, as it showed potential in suppressing lung cancer, breast cancer, gastric cancer, liver cancer, glioma, melanoma, and tongue cancer, etc., as summarized in Table 1 and Figure 2. The full potential of lidocaine in sensitizing other chemotherapeutics and combating cancer remains to be explored.

**TABLE 1:** Summary of lidocaine in cancer treatment.

Category	Applications	Mechanisms/Targets	References
**Chemosensitizer**	Sensitizing cisplatin	DNA demethylation PTEN/PI3K/AKT and PDCD4/JNK pathways Metastasis inhibition	[Bibr B60][Bibr B125][Bibr B27]
	Sensitizing 5-FU	PI3K/AKT and Smad pathways ABC transporters regulation	[Bibr B116][Bibr B132]
	Sensitizing mitomycin C Sensitizing hyperthermia	Unreported Cell cycle arrest and HSPs regulation	[Bibr B127][Bibr B86]
**Anticancer agent**	Lung cancer	MMP-9 or AKT/FAK pathways GOLPH2	Piegeler et al., 2015; Zhang et al., 2017
	Breast cancer	CXCL12/CXCR4 signaling Anti-inflammatory and anti-angiogenic effects Metastasis inhibition DNA demethylation	[Bibr B19][Bibr B40][Bibr B11][Bibr B65]
	Liver cancer	p53 inhibition Cell cycle arrest and apoptosis induction CPEB3	[Bibr B43][Bibr B120][Bibr B67]
	Gastric cancer	Up-regulation of miR-145 Mitochondria-mediated apoptosis pathway ERK1/2	[Bibr B100][Bibr B129][Bibr B126]
	Colorectal cancer	miR-520a-3p Apoptosis induction Cell cycle arrest	[Bibr B85][Bibr B107][Bibr B8]
	Melanoma	Apoptosis induction Ki-67 Anti-angiogenesis	[Bibr B45][Bibr B12][Bibr B29]
	Glioma	TRPM7 TRPV1 TRPV6	[Bibr B56][Bibr B68][Bibr B39]
	Tongue cancer	EGFR Mitochondrial respiration inhibition	[Bibr B91][Bibr B134]

Currently, lidocaine is undergoing multiple clinical trials for the treatment of various cancer types and cancer-related symptoms, including an early phase I study for the prevention of recurrence of pancreatic cancer upon lidocaine (intravenous); a phase I for the prevention of recurrence of gastric carcinoma, colon cancer, rectal carcinoma, and liver cancer by injection of 0.5% lidocaine in normal saline to block peripheral nerve; and a phase III for better outcome of breast cancer via dermal infiltration of 2% lidocaine in incision site and regional breast and axillary areas (ClinicalTrials.gov Identifier: NCT04048278, NCT03134430, and NCT00938171). Some of these clinical trials are summarized in Table 2. Most of these clinical trials are in the early stages, the applications of lidocaine in cancer treatment are yet to be proved.

**TABLE 2:** Several clinical trials using lidocaine in cancer treatment^a^.

Phase/Year initiated	Identification code	Indications	Status/Results
Early Phase I/2019	NCT04048278	Pancreatic cancer	Recruiting/Not reported
Phase I/2017	NCT03134430	Cancer recurrence	Active/Not reported
Phase III/2009	NCT00938171	Breast cancer	Completed/Unrevealed
Phase II/2020	NCT04295330	Pain management in liver cancer	Recruiting/Better efficacy and safety were observed
Phase I/2019	NCT04162535	Efficacy and prognosis of colorectal cancer	Recruiting/Not reported
Phase II/2016	NCT02839668	Angiogenesis in breast cancer	Completed/Unrevealed
Early Phase I/2016	NCT02786329	Chronic pain, cancer recurrences	Recruiting/Not reported
Phase I/2018	NCT03530033	Muscle tumor in thyroid surgery	Recruiting/Not reported

*^*a*^Details available at ClinicalTrials.gov.*

In addition, as per the safety profile, lidocaine not only shows well-tolerant toxicity (Yang X. et al., 2018), selectivity in killing cancer cells over normal cells (Chamaraux-Tran et al., 2018), but also may alleviate cisplatin-induced body loss in the animal studies (Gao et al., 2018).

As reviewed in this paper, we noticed that the applied doses/concentrations of lidocaine varied when it came to different effects. For in vitro studies, lidocaine may work as chemosensitizer at relatively lower concentrations—for example, lower than 200 μM—while it requires a higher concentration to kill cancer cells, from 8 mM (Sun and Sun, 2019) up to 4 M (administered topically), which is unachievable in clinical practice, in killing tongue cancer cells (Sakaguchi et al., 2006). There is also research showing that under clinically relevant concentrations (0.1–200 μM), lidocaine exhibited anticancer efficacies. The plasma concentration of local anesthetics is at low *μ*M, ranged from 2.5–10 μM, suggesting a suitable administrative method, such as local infiltration or administering topically, should be taken into account when used for killing cancer cells (Li R. et al., 2018; Zhu et al., 2020). More efforts, including the pharmacokinetics profile in vivo, are needed to verify its campatibal dose/concentration in certain treatments that are achievable in clinical practice.

Although the anticancer efficacies can be confirmed in various cancers, its exact mechanisms/targets remain unclear. As summarized in Table 1 and Figure 2, the reported targets/mechanisms include MMP-9 (Piegeler et al., 2015), ERK (Chen et al., 2019), TRPV6 (Jiang et al., 2016), and VEGF/VEGFR2 (Gao et al., 2019), as well as the regulation of epigenetics (Li et al., 2014), mi-RNA (Qu et al., 2018; Sui et al., 2019; Yang Q. et al., 2019), inhibition of metastasis (D’Agostino et al., 2018; Johnson et al., 2018; Sun and Sun, 2019), inhibition of inflammation (Freeman et al., 2019), impacting mitochondrial metabolism (Okamoto et al., 2017; Gong et al., 2018), the regulation of reactive oxygen species (ROS) (Okamoto et al., 2016), etc. Importantly, as an ion channel regulator, lidocaine may exert its anticancer effects via regulation of other channels or membrane potential, such as mitochondrial membrane potential (MMP), which may lead to the decrease of MMP, finally resulting in the mitochondria-related apoptosis (Li et al., 2014; Lu et al., 2016; Ye L. et al., 2019), providing valuable information for its targets identification. However, further studies are warranted to confirm its exact targets in different cancer types.

The information obtained about lidocaine could direct researchers to screen more local anesthetics for their cancer-inhibiting capabilities. Indeed, many local anesthetics have been confirmed to possess anticancer efficacies, such as morphine (Kim et al., 2016; Chen et al., 2017; Nishiwada et al., 2019), although it demonstrates contrasting results in cancer treatment (Harper et al., 2018; Tuerxun and Cui, 2019); procaine (Ma et al., 2016; Li C. et al., 2018; Li Y.C. et al., 2018); ropivacaine (Piegeler et al., 2015; Gong et al., 2018; Wang et al., 2019; Yang J. et al., 2019); bupivacaine (Xuan et al., 2016; Dan et al., 2018); and levobupivacaine (Jose et al., 2018; Li et al., 2019); as well as propofol (Xu et al., 2018; Kang et al., 2019; Zheng et al., 2019), etc.

As a chemosensitizer, certain combination strategies via different formulations can be designed and developed. Certain formulations composed of lidocaine with various anticancer drugs have been developed and they exhibit positive results in cancer treatment (Tabrizi and Chiniforoshan, 2016, 2017).

Furthermore, these studies also provide several leading scaffolds, namely, lidocaine, ropivacaine, and propofol, that may undergo structural modification to improve their activities and to avoid off-target effects. Examples include the palladium(II) complexes coordinated with lidocaine and phenylcyanamide derivatives as ligands (Tabrizi and Chiniforoshan, 2016, 2017), which have shown better antiproliferative effects than cisplatin. Series of propofol-derived analogs or derivatives have been synthesized and tested in various cancer cells and some of them exhibited favorable anticancer potency (Khan et al., 2011, 2012, 2013). These studies further strengthen the possibilities and potentials of using local anesthesia as anticancer agents; however, the outcomes can’t be overestimated until the structure–anticancer activity relationships are deciphered. Given that propofol, lidocaine, and ropivacaine possess different structures and pharmacophores, they may also exert their anticancer effects via different mechanisms. Further applications of these anesthesia in clinic will sure benefit from further studies of the exact action modes.

## Conclusion

Lidocaine, via various mechanisms, could suppress cancer growth *in vitro* and *in vivo*, providing valuable information for its further application in cancer treatment and for new drug discovery.

## Author Contributions

YX, PX, and QC conceived the idea. DZ, QC, and RI wrote the manuscript. LW, YX, and PX revised the manuscript. All authors contributed to the article and approved the submitted version.

## Conflict of Interest

The authors declare that the research was conducted in the absence of any commercial or financial relationships that could be construed as a potential conflict of interest.
